# The Semiaquatic Nematoceran Fly Assemblages of Three Wetland Habitats and Concordance with Plant Species Composition, a Case Study from Subalpine Fennoscandia

**DOI:** 10.1673/031.011.0135

**Published:** 2011-03-24

**Authors:** Jukka Salmela

**Affiliations:** Department of Biology, Zoological Museum, FI-20014 University of Turku, Finland

**Keywords:** Lapland, Finland, Tarvantovaara wilderness area, aapa mires, springs, headwater streams, dipteran diversity

## Abstract

Semiaquatic flies (Diptera, Nematocera) are an ecologically important and species rich group of insects within the boreal and arctic biomes. Community structure, species richness and abundance of semiaquatic flies were studied in three habitat types (aapa mires, springs and headwater streams), totaling 19 study sites, within the subalpine ecoregion of northern boreal Finland. Concordance of semiaquatic fly species composition with plant assemblages (higher plants and mosses), and geographical and environmental distance matrices were also studied. The collected insect material consisted of 94 species and 9038 specimens. According to non-metric multidimensional scaling ordination (visual inspection), multi-response permutation procedure and analysis of similarity tests, fly assemblages of aapa mires were clearly different from those of springs and headwater streams, but no differences were found between spring and headwater stream assemblages. The cumulative number of species was highest in headwater streams. Alpha diversity varied within the habitat types but was generally highest among headwater streams. Semiaquatic fly communities of headwater streams were the most abundant (number of specimens) and their rank-abundance distributions were relatively skewed; assemblages of aapa mires were less abundant and rather even. Community composition of combined plant material (219 taxa), higher plants (116 taxa) and mosses (103 taxa) were all in concordance with the flies; the strongest matrix correlation was found between higher plants and flies (Mantel test). The influence of geographical distance of the study sites to species composition was statistically significant but rather weak; instead, much stronger concordance was noted with environmental variables (Mantel test). Plants, especially higher plants, may be potential surrogates for semiaquatic fly assemblage composition. However, more studies of community concordance in a larger geographic area and within one habitat type are needed.

## Introduction

Species composition of terrestrial communities is a consequence of several hierarchical and interacting factors. The regional species pool is a result of climate, geology, historical events and evolutionary processes. Further, dispersal ability and habitat selection of the species affect the identity of the potential colonizers and interspecific interactions facilitate or inhibit the co-occurrence of the species in a given locality (Morin 1999). Assemblages (*sensu* Fauth et al. 1996) are phylogenetically related members of the biological community. Most often taxonomically related assemblages rather than whole communities are surveyed, because inventories of small bodied and species rich taxa need huge scientific effort (Lawton et al. 1998) and are beyond possibility in most cases. Ecological investigations of lesser known groups (such as many insects, nematodes and fungi) are needed, because in most ecosystems diversity patterns and composition of two or more taxonomic groups are seldom or only weakly correlated (Gaston and Spicer 2004; Cox and Moore 2005).

Positive cross-taxon concordance in either alpha or beta diversity refers to a situation in which one (or more) taxonomic group could be used as a surrogate for other groups; congruence in assemblage structure is a highly interesting aspect in applied ecology, such as biomonitoring (e.g. Carlisle et al. 2008; Mykrä et al. 2008) and nature conservation (e.g. Su et al. 2004; Bilton et al. 2006). There are, however, rather conflicting results about community concordance in different habitats or regions. For example, in two rather large studies of terrestrial habitats in the southern hemisphere, no support was found for the use of a reliable surrogate group (van Jaarsveld et al. 1998; Lawton et al. 1998). Instead, in a study of meadow communities in North America, a positive concordance in assemblage structure, but not in species richness, was reported (Su et al. 2004). In the boreal zone, biotic groups of streams and rivers showed concordant patterns only if the spatial gradients were long enough (Paavola et al. 2006). To clarify, different lotic groups responded differently to environmental variables within a scale of a catchment, but between-ecoregion concordance was probably caused by biogeographic factors (post-glacial history, latitudinal distribution patterns) (Paavola et al. 2006). Concordant patterns in assemblage structure have most often been studied with relatively well known taxonomic groups, such as fishes, benthic macroinvertebrates, diatoms and macrophytes in freshwater ecosystems (e.g. Paavola et al. 2006; Grenouillet et al. 2008) and plants, butterflies, beetles and birds in terrestrial ones (Blake et al. 2003; Su *et al*. 2004; Similä et al. 2006; Davis et al. 2008). Notable exceptions are e.g. studies by Sääksjärvi et al. (2006) on parasitoid wasps and tropical plants and Murray et al. (2006) on curculionid beetles, lichens, bryophytes and higher plants. Thus, there is need for concordance studies gathering poorly known taxonomic groups and habitats.

In terms of species richness, habitat associations and functional feeding groups, semiaquatic flies (Diptera, Nematocera) are a diverse group of insects, of which most species are dwellers of wetlands and small water bodies (Salmela 2004; Salmela et al. 2007; de Jong et al. 2008; Wagner et al. 2008). Many semiaquatic fly species are stenotopic, that is, their life cycles are bound to rather narrow habitat niches, such as springs, rich fens, headwater streams, fruiting bodies of fungi or decaying wood (e.g. Alexander 1920; Brindle 1967; Salmela et al. 2007; Hancock et al. 2009). Hence, semiaquatic flies are considered to have great potential for biomonitoring, conservation and assessment of freshwater habitats or other wetlands (Chadd and Extence 2004; Salmela 2008). Semiaquatic flies belong to 12 families and 424 semiaquatic fly species are currently known from Finland (Salmela 2010). Within the semiaquatic flies, craneflies *sensu lato* (Tipulidae, Limoniidae, Pediciidae, Cylindrotomidae) are the most species rich group (330 spp), followed by moth flies (Psychodidae, 57 spp); other families are relatively species poor, each of them having 1-15 species (Ptychopteridae, Dixidae, Thaumaleidae, Synneuridae, Canthyloscelidae, Pachyneuridae, Pleciidae). Semiaquatic flies have been rather neglected and poorly known in Finland up till the beginning of 2000's; studies dealing with the ecology of the species and community parameters such as diversity, abundance and species turn-over, have been very few in number (see Salmela 2008 for a short review about northern Fennoscandia).

Within the northern boreal vegetation zone in Finland, the local richness (alpha diversity) of semiaquatic flies does not decrease with increasing latitude, but a remarkable change occurs in the regional species pool at the border of coniferous forests and subalpine ecoregion (Salmela 2008). On a large scale, within the extent of northern boreal zone, species composition of semiaquatic flies are influenced by biogeographical factors and local conditions, especially factors which are connected to hydrogeology (e.g. ground water influence, mineral composition of the bedrock) and to the dominating habitat type (Salmela 2008). The variation of species richness and community composition on a smaller spatial scale (i.e. within a drainage area or ecoregion) and the factors influencing them, are insufficiently known. In addition, the community concordance of semiaquatic flies with other biotic groups, like plants, is not well known.

The main goal of the present study is to investigate the variation in assemblage composition and species richness of semiaquatic flies in the subalpine ecoregion, northernmost Finland. Specifically, this study is trying to seek out patterns in the following issues: (i) what is the amount of variation in the species richness and assemblage composition in three habitat types (aapa mires, springs, headwater streams) and (ii) to study the correlation of semiaquatic flies with plant species composition (higher plants and mosses), environmental variables and geographical distance (spatial autocorrelation). It has recently been shown that terrestrial arthropod assemblages are well predicted by plant species composition; in fact, plants proved to be better determinants of arthropod species composition than environmental variables or physical vegetation structure (Schaffers et al. 2008). It is thus highly interesting to compare the performance of environmental variables and plant species composition as surrogates for semiaquatic fly assemblage composition. This study is part of the national survey of semiaquatic flies which aims to assess the redlist status and distribution patterns of the species, and develop tools for the conservation and assessment semiaquatic fly communities (J. Salmela, unpublished data).

## Materials and Methods

### Study area

The study area is located in northernmost Finland (ca. 68°35′N 22°40′E), biogeographical province of Lapponia enontekiensis, belonging to the northern boreal vegetation zone and to its subzone, the subalpine ecoregion (Lindholm and Heikkilä 2006). In some publications this subzone is also called the “Arctic-alpine” ecoregion (e.g. Soininen et al. 2004). The subalpine ecoregion is characterized by virtual lack of coniferous forests; the tree line is formed by mountain birch, *Betula pubescens* ssp. *czerepanovii* (Orlova) Hämet-Ahti (Fagales: Betulaceae) and large districts are treeless fell areas. The study area is part of the Tarvantovaara wilderness area (67030 ha), its landscape is dominated by mountain birch forests, mires and fells. This district is practically lacking any year-round settlements or public roads. The altitude in the area ranges between 360 to 630 m a.s.l. and the bedrock is composed of variety of rocks, including granodiorite, quartzite, metavolcanites and gabbro (Table 1). The climate is continental, that is, relatively low annual precipitation (ca. 450 mm) and cold winters (mean temperature of January ca. -16° C) are prevailing (Piirainen 2002; Tikkanen 2006). Evaporation does not exceed precipitation during the thermal growing season (105–115 days ≥ 5° C, Piirainen 2002), and thus, runoff from the drainage areas is relatively large despite of low amount of precipitation. This phenomenon is strikingly seen in the occurrence of numerous sloping fens, which indicate high moisture level.

### Selection of study sites and measurement of environmental variables

**Figure 1.**
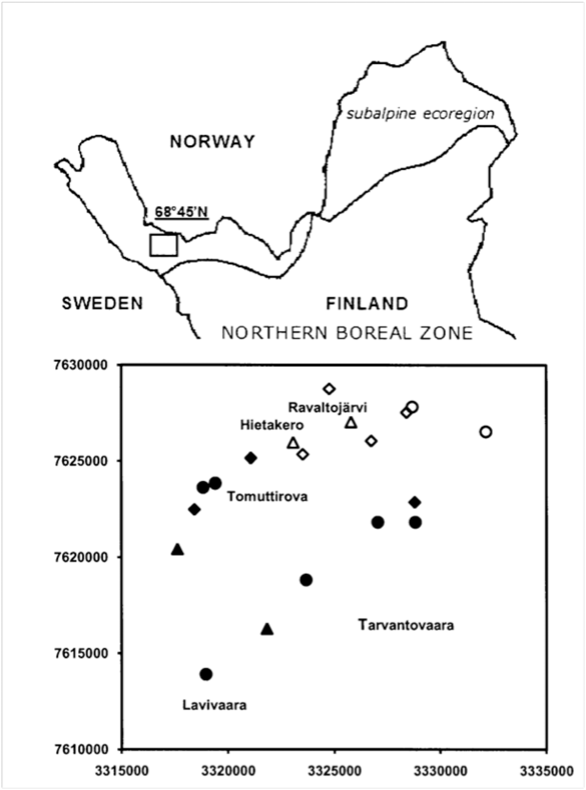
Location of the study area in Finland, subalpine ecoregion (above). Graphical representation of the study sites (below). Vertical and horizontal axes are north and east coordinates (Finnish uniform grid 27° E coordinate system, coordinates are shown in 5 km intervals). Circle=aapamire, triangle=spring and diamond=headwater stream. Filled symbols are located between altitudes of 410–450 m a.s.l. (birch zone) and open symbols are located in the tree-less fell area (460–540 m a.s.l.). Lavivaara, Hietakero and Tarvantovaara are fells, Tomuttirova is a glacifluvial formation (esker) and Ravaltojärvi is a lake. High quality figures are available online.

A total of 19 study sites were selected; eight of these were aapa mires, four were springs and seven were headwater streams (Table 1, Figure 1). *Aapa mires* in the subalpine ecoregion are minerotrophic fens, characterized by flarks (i.e. inundated, mudbottom or carpet level vegetation, mainly inhabited by horizontally growing bryophytes, see Rydin and Jeglum 2006 for terminology) surrounded by irregular pattern of strings, (hummock-level vegetation, dominated by dwarf shrubs). Most of the studied aapa mires were poor and intermediate rich in their floristic composition, that is, they were dominated by *Sphagnum* sp. and *Warnstorfia procera* (Renauld and Arnell) Tuomikoski (Hypnales: Amblystegiaceae) mosses. Some aapa mires were rich fens, i.e. they were characterized by *Scorpidium* mosses. *Springs* are points of emerging groundwater, usually stable in terms of water temperature and discharge. One of the studied springs had a minerogenous bottom of the spring brook while in other sites the outflow took place over soft bottom or moss vegetation (e.g. *Warnstorfia exannulata* (W.Gümbel) Loeske, *Philonotis fontana* (Hedwig) Bridel). Springs were quite sharply separated from the surrounding biotopes (birch forests, alpine heaths and mires). Unfortunately, only four springs were studied. The original plan was to include more springs, but suitable sites were either not available in certain areas or not accessable due to difficult terrain. *Headwater streams* are small lotic waters located in the upper reaches of the catchment. In relation to springs, headwater streams are much more variable in their discharge and water temperature. The headwater streams of this study were first or second order streams, characterized by small drainage areas (92–1004 ha) and minerogenous bottoms (sand, gravel, stones and boulders); mosses such as *Fontinalis dalecarlica* Schimper and *Hygrohypnum alpestre* (Sw. Ex Hedwig) Loeske were common inhabitants on the submerged and emergent stony substrates of the streams. Riparian zones of headwater streams were rich in plant species, which included species typical for mires, herb-rich forests and alpine meadows.

**Table 1.**
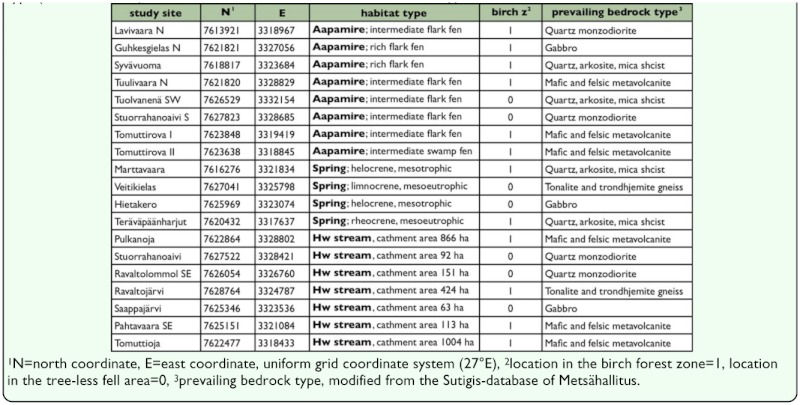
Study sites in the Tarvantovaara wilderness area, subalpine ecoregion, Finland, their location (coordinates), habitat type (hw= headwater), location in the fell area/birch zone and bedrock type.

The study sites were chosen beforehand to cover gradients in the bedrock composition (siliceous —base rich stones) and altitude (birch forest — fell areas). The shortest distance between the study sites was 0.4 km and the longest distance 16.9 km, average distance between the sites was 7.4 km; the study sites were located in an area ca. 225 km^2^. Malaise traps (see below) were set during the first visit in the beginning of June (8–12 June 2009). In aapa mires, the traps were placed in the immediate vicinity of flarks. In springs and headwater streams, the traps were set over the flowing water or in the bank area as close as possible to the spring pool or flowing water. Each trap formed the centre of the study site which was a 10 × 30 m rectangle. The following environmental variables were measured from the study sites: tree basal area (i.e. the cross-sectional area over the at breast height, m^2^/ha), coverage (%) of different moisture levels and soil types (water, flark, intermediate, hummock, total peatland area and minerogenous substrates), water temperature (GEFU digital thermometer, precision +/- 0.1) and pH (pHep-portable instrument, precision +/- 0.1); water temperature was measured three times (June, July, end of August) and pH in July. Finally, altitude was measured from topographic maps and exact location with a GPS-navigator (Garmin etrex, precision +/- 8 m).

### Sampling of adult insects and identification of plant species composition

One Malaise trap was placed in each study site. Malaise (length 110, height 140, width 70 cm) is a trap model made of cloth (black sides, white cover) and is suitable for collecting low-flying insects, especially efficient for dipterans, hymenopterans, trichopterans and plecopterans. Based on experience of ten years and >400 trapping sites, no protected invertebrates or any vertebrates have been caught by the traps. The traps were set in the beginning of June (8–12 June 2009), collecting jars were emptied in the middle of July (18–24 July) and in the end of August (23–27 August); traps also were removed from the field during this last occasion. A solution of 50 % ethylene glycol + few drops of detergent was used as a preservative in the traps. The collected material was stored in 80 % ethanol. The semiaquatic fly specimens were sorted from the material in the laboratory and were identified to species level. During the last field trip in August sweep net samples (ca. 15 min collecting effort in each site) were taken and this material was later combined with the Malaise trap material (i.e. final species × sample units matrix). Literature for the identification of semiaquatic flies comes from hundreds of sources, too numerous to be referred here. Labeled museum samples (in 80 % ethanol, 2 ml plastic tubes) of most species are deposited in the Zoological Museum, University of Turku, Finland (ZMUT).

Higher plants (Tracheophyta) and bryophytes (mosses and liverworts) were identified during the field trip in July; the aim was to detect all plant taxa present, to species or genus level. For each site the plant species composition was inventoried (presence/absence) from the 10 × 30 m study plots. In the relatively monotonous aapa mires the inventories were performed within 30–45 minutes and in the structurally more complex lotic sites ca. 60–90 minutes. A hand lens (30×) was used in the field and samples were taken for later identification in the laboratory. Nomenclature of the higher plants and bryophytes follows Hämet-Ahti et al. (1998) and Ulvinen and Syrjänen (2009), respectively (Appendix 2).

### Data analysis

One-way ANOVA was used to test for differences between the mean values of the variables (species richness, abundance etc.) from the studied habitats (aapa mires, springs, headwater streams). In pair-wise *post hoc* comparisons, Tukey's HSD test was used. In order to validate the use of parametric method, the homogeneity of the variances and normal distribution of the variables were assessed with the tests of Levene and Shapiro-Wilk, respectively. If these assumptions were not satisfied, the non-parametric Kruskal-Wallis test was used with the Mann-Withney U-test in pairwise comparisons. Correlations between the species richness of semiaquatic flies, plant species richness and environmental variables were studied by using nonparametric Spearman rank correlation coefficient. The chi square test (χ^2^) was used to examine whether there were differences in the number of occurrences of noteworthy species (red-listed and national responsibility species) in the studied habitats.

Semiaquatic fly community composition of the study sites were examined and viewed with a number of multivariate methods. NMS (non-metric multidimensional scaling) is an ordination method, in which the original ranked distances (based on distance measure) of the sample units in the *p* dimensional species space are forced to a reduced, *k* dimensional ordination (Legendre and Legendre 1998; McCune and Grace 2002). Spearman correlation coefficients were calculated between the ordination's coordinates of the sampling units and environmental variables. Significant correlations are instrumental in the assessment of which variables are associated with the position of study sites on different dimensions of the ordination. McCune and Grace (2002, pp. 107–108) questioned whether it is appropriate to present *p*-values in this connection because coordinate points of the sampling units along the dimensions are not independent variables. However, it is possible to describe and interpret the main directions of variation along the dimensions of the ordination.

MRPP (multi-response permutation procedure) is a non-parametric method for testing the null hypothesis no difference in assemblage composition of two or more *a priori* defined groups (McCune and Mefford 1999). The within-group homogeneity of each group (observed δ) is compared to a random arrangement of the sampling units (expected ο) and this difference is the effect size *A* (chance-corrected within-group agreement). *A*=1, if all sampling units within a group are identical and *A*=0, if the within group variation in the species composition equals expectations by chance. The statistical inference (*p*-value) is based on a comparison of frequency distribution of randomized values and observed δ. ANOSIM (analysis of similarity) is a non-parametric method for testing whether there are differences in the assemblage composition of two or more *a priori* defined groups (Legendre and Legendre 1998). Similarity (or distance) is calculated between all sample units within each group and afterwards these similarities are rank-transformed (i.e. sample units, which have highest resemblance, rank-order is 1). The same procedure is performed with betweengroup similarities. Test value *R* is based on the remainder of the between-group and within-group mean rank-order similarities (see Legendre and Legendre 1998 for details) and p-value is based on permutation (10000 permutation were used). *R* values may range between 1 and 0: the higher the value, the more differentiated assemblages between the groups.

In the above mentioned multivariate methods (NMS, MRPP, ANOSIM) log (*x*+1) transformed data matrices of semiaquatic flies and Bray-Curtis metric were used. Logarithmic transformation was seen as necessary, because the abundances (number of specimens) were quite variable between the study sites. This transformation reduces the importance of the most numerous species and thus, gives more weight to less numerous species.

The Mantel test was used to examine concordance of semiaquatic flies with (i) higher plants, (ii) bryophytes, (iii) combined plant material (higher plants + bryophytes), (iv) geographical distance of the study sites and (v) environmental variables. The Mantel test is used to test the null hypothesis of no relationship between two distance matrices, i.e. the test evaluates linear correlation between two distance matrices. Each matrix is calculated from a different set of variables, measured for the same sample units (study sites) (Legendre and Legendre 1998; McCune and Grace 2002). The test value r_M_ is analogous to the Pearson correlation coefficient (range -1 and 1). Statistical significance is calculated by permutation (9000 permutations were used). Presence/absence data matrix of semiaquatic flies was used because plant species composition was measured to the accuracy of presence/absence; it is recommended to use the same matrix transformation in the comparisons of two distance matrices (Heino 2008). Because geographical and environmental distance matrices (Euclidean distance in both) showed positive Mantel correlations with semiaquatic fly distance matrix, partial Mantel tests were also used in the semiaquatic fly × plant matrices correlations. Partial Mantel test can be used to examine the relationship between two resemblance matrices while eliminating the linear effect of third matrix (Legendre and Legendre 1998).

The investigation of species accumulation in the habitat types was based on the assessment of the number of samples (=number of study sites per habitat type) and number of individuals. Because there was only one trap per study site, it was not possible to use means and SDs to study the effect of different number of traps on the species accumulation within a single site. Instead, the cumulative number of species was calculated for the combined material for each habitat type (Mao-Tau method, see Colwell 2009). The rarefaction method was used for the calculation of individual based accumulation curves. Rarefaction is instrumental if the sampling efficiency between the study sites has varied and the samples should be standardized to a given number of specimens (Krebs 1998).

Non-parametric species richness estimators (Jackknife1, Jackknife2, Chao1, Chao2) were used to evaluate the total number of species (observed + unseen species) in the combined material for all study sites (local species pool) and for habitat types (local species pool within each habitat type). These estimators are based on the assumption that the observed species richness is lower than the true richness of the study sites (e.g. Colwell and Coddington 1994; McCune and Grace 2002; Magurran 2004; Colwell 2009). These estimators are best suited for an analysis of such communities, in which a given study site has several plots, quadrats, traps or other sample units of similar size (Krebs 1998; Magurran 2004). In this study these estimators were seen as appropriate because at each site the sampling intensity was of the same magnitude and the combined material from the habitat types most probably covered >50 % of the estimated number of species (Ulrich and Ollik 2005).

Mean values of the Simpson diversity index (1-D) were calculated for the habitat types. This index is calculated as
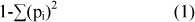

where p_i_ is the proportion of the species *i* of the total number of specimens in a community (Krebs 1998). The diversity index is linked to the probability that two randomly chosen specimens belong to different species. This index was used together with the rank-abundance distributions (Whittaker plots) and the percentages of the most abundant species, to evaluate how skewed the species' abundances are among the habitat types.

MRPP test was calculated by using PC-ORD 5.0 (McCune and Mefford 1999), species accumulation curves and non-parametric species richness estimators by using Estimates 8.2.0 (Colwell 2009) and Mantel tests by using Fstat 2.9.3.2 (Goudet 1995) and other analyses by using PAST 1.94b software (Hammer et al. 2001).

## Results

### Assemblage composition of semiaquatic flies, relationships with plant species composition, environmental variables and geographical distance

**Figure 2.**
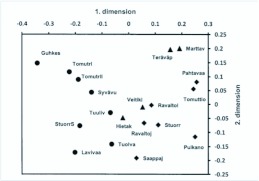
NMS ordination along first and second dimensions of the 19 study sites, based on log (*x*+*l*) transformed distance matrix (Bray-Curtis) of semiaquatic flies. Symbols: circle=aapamire, triangle=spring and diamond=headwater stream. Names of the study sites (see Table 1) are abbreviated. Correlation coefficients between ordination scores and environmental variables are presented in Table 5. High quality figures are available online.

The collected material of adult semiaquatic flies was composed of 9038 specimens of 94 species (Appendix 1). A total of 1592 specimens (exx.) and 62 species (spp.) were found from aapa mires, the corresponding figures for the spring and headwater streams are 1288 (exx.), 49 (spp.) and 6158 (exx.), 82 (spp.), respectively. According to NMS ordination (Figure 2), headwater streams are clearly separated from the aapa mires, springs are located closer to headwater streams but do not form their own separable group. The distribution of the study sites along the first dimension correlates significantly with several environmental variables which are related to the conditions typical for mires (positive correlation) and lotic waters (negative correlation); the second dimension is correlated only with altitude (Table 3). The third dimension correlates weakly with the same parameters as the first dimension, and this dimension is practically redundant with the first dimension (Table 3). In other words, the arrangement of the study sites along the first dimension is mirroring a gradient of hydrological conditions and the second gradient discriminates the sites according to an altitudinal gradient.

**Table 2.**
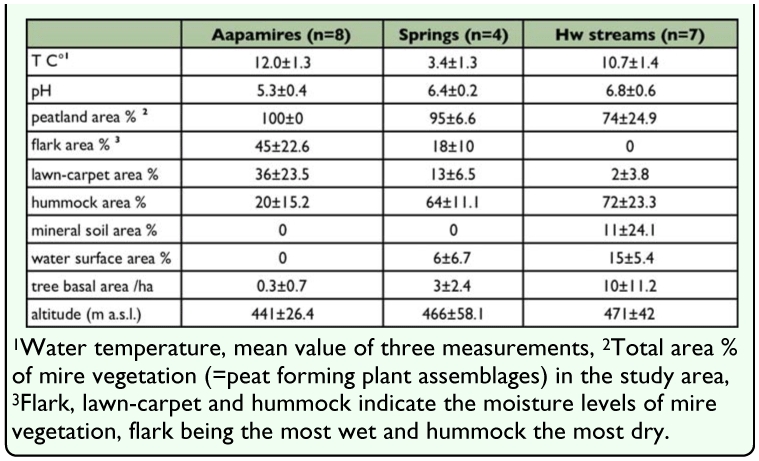
Environmental variables measured from the study sites and their mean (±standard deviation) values in the habitat types.

**Table 3.**
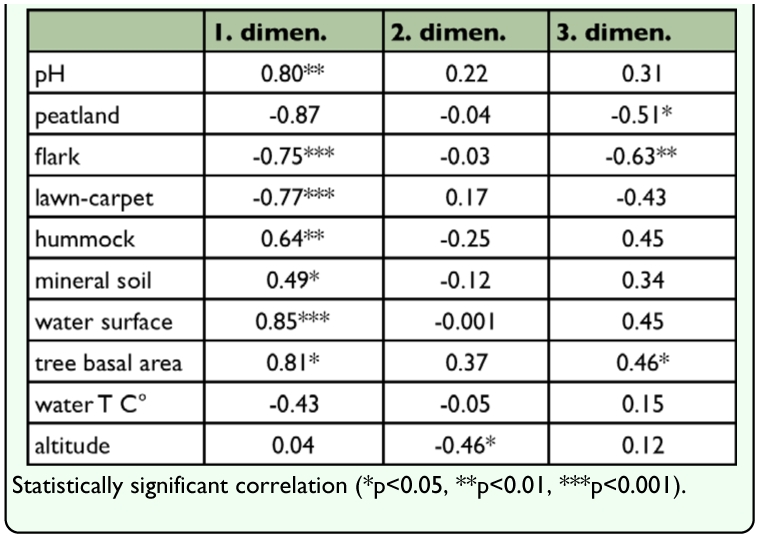
Correlation cofficients (Spearman) and the associated pvalues between the NMS ordination scores of semiaquatic flies (dimensions 1–3) and environmental variables.

**Table 4.**
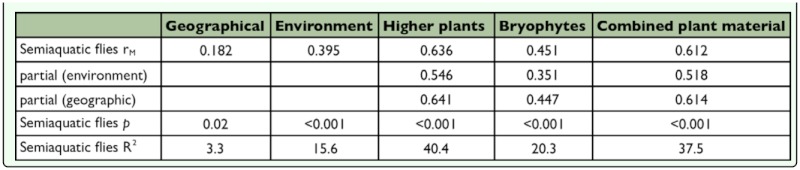
Mantel r_M_ test values, *p*-values and coefficient of determination (R^2^ %, variance explained) for two-matrix correlations of semiaquatic flies (SF) between geographic distance, environmental distance and between distance matrices of higher plants, bryophytes and combined plant material. Partial Mantel test values (i.e. two assemblage matrices controlled against the effect of environmental and geographical distance matrices) are given.

According to MRPP test, the assemblages of the habitat types were significantly different (*A*=0.326, *p*<0.001). In pair-wise comparisons aapa mires differed from springs (*A*=0.218, *p*=0.002) and headwater streams (*A*=0.341, *p*<0.001), but no difference was found between the assemblages of springs and headwater streams (*A*=0.076, *p*=0.11). The result of the ANOSIM test was in accordance with the MRPP, since the *a priori* classification of the assemblages was highly significant (*R*=0.532, *p*<0.001). Pair-wise comparison revealed that aapa mires differed from springs (*p*=0.021) and headwater streams (*p*<0.001), but no difference was found between springs and headwater streams (*p*=0.313).

**Figure 3.**
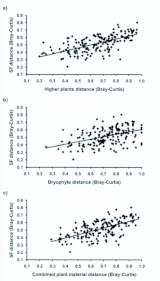
Correlations between assemblage dissimilarities (Bray-Curtis) of a) semiaquatic flies and higher plants, b) semiaquatic flies and bryophytes and c) semiaquatic flies and combined plant material (higher plants + bryophytes). Coefficients of determination (R^2^) are given in the Table 4. High quality figures are available online.

**Figure 4.**
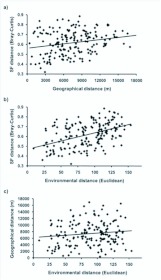
Correlation between a) semiaquatic fly assemblage dissimilarity (Bray-Curtis) and geographical distance (km), b) semiaquatic fly dissimilarity and environmental distance (dissimilarity, Euclidean) and c) geographical distance and environmental distance. Coefficients of determination (R^2^) are given in the Table 4 (except for Figure 4C, which is 0.014). High quality figures are available online.

According to Mantel tests, the community concordance of semiaquatic flies with higher plants (r_M_=0.636), bryophytes (r_M_=0.451) and combined plant material (rM=0.612) were highly significant (Table 4, Figure 3); in other words, distance matrices of semiaquatic flies and plants were positively correlated. Partial Mantel statistics (i.e. the controlling effect of environmental variables and geographical proximity) had an only minor effect on the matrix correlations (Table 4). The association of semiaquatic fly assemblages and geographical distance between the study sites was positive but rather weak (r_M_=0.221). Instead, correlation between environmental variables and semiaquatic flies was higher (r_M_=0.452) (Table 4, Figure 4). Matrix correlation between geographical distance and environmental variables of the study sites was non-significant (Mantel test: r_M_=0.063, *P*=0.405). However, higher plant species composition (R^2^=40.4 %) was superior as an explanatory variable of semiaquatic fly species composition over bryophytes (R^2^=20.3 %), environmental variables (R^2^=20.4 %) and geographical distances (R^2^=4.9 %) (Table 4, Figures 3 and 4).

**Table 5.**
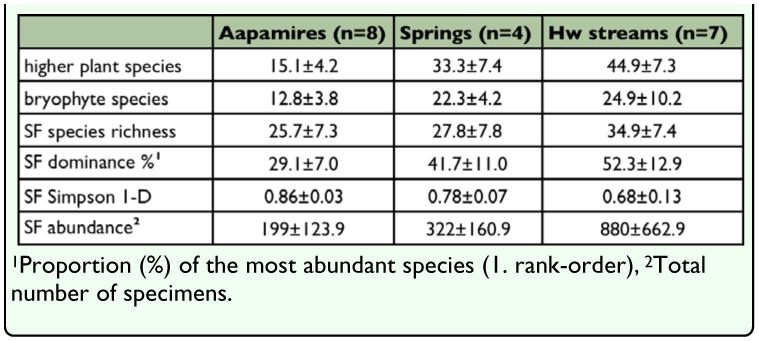
Mean (±SD) species richness of higher plants, bryophytes and semiaquatic flies (SF) in the habitat types. Mean values are also presented for diversity and abundance variables of semiaquatic flies.

### Patterns in species richness and abundance of semiaquatic fly communities, rankabundance distributions and estimation of species richness

**Figure 5.**
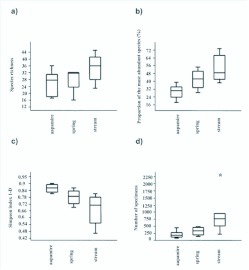
Box plots (median, quartiles and range of values) of semiaquatic fly a) species richness b) proportion (%) of the most abundant species, c) Simpson diversity index and d) abundance (total number of specimens) in the studied habitat types (aapa mires n=8, springs n=4, headwater streams n=7). High quality figures are available online.

The species richness of semiaquatic flies varied in the habitat types and was generally highest amongst headwater streams (Table 5, Figure 5a), near-significantly (ANOVA F=2.9, df=2, *p*=0.08). The habitat types studied differed in the proportion of their most abundant species (ANOVA F=10.3, df=2, *P*=0.0013, square-root transformed), pair-wise *post hoc* comparisons indicate a significant difference between aapa mires (lowest dominance of the most numerous species) and headwater streams (highest dominance of the most numerous species) (*p*=0.0009) (Table 5, Figure 5b). Similar results were obtained by using a diversity index: mean values of the Simpson (1-D) index were different between the sites (Kruskal-Wallis H=11.2, *p*=0.004) (Table 5, Figure 5c). Pair-wise comparisons show that the index value was higher among aapa mires than headwater streams (Bonferroni corrected *p*=0.006). Finally, mean values of the raw-abundance (number of specimens) differed between the habitats (ANOVA F=8.7, df=2, *p*=0.0028, log transformed) (Table 5, Figure 5d); according to pair-wise comparisons the average number of specimens in aapa mires was lower than that of headwater streams (*p*=0.02). Correlations (Spearman rank-correlation coefficient) of the semiaquatic fly species richness with the combined plant material (r=0.4, *p*=0.09) and higher plant species richness (r=0.4, *p*=0.08) were positive but not significant; correlation with bryophyte richness was also positive but also not significant k (r=0.35,/*p*=0.15).

**Figure 6.**
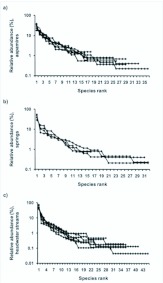
Rank-abundance distributions of semiaquatic fly assemblages in a) aapa mires, b) springs and c) headwater streams. High quality figures are available online.

The rank-abundance distributions of the semiaquatic fly communities among the habitats were quite similar, resembling truncated log-normal type distribution (Figure 6a–c). A typical feature of the assemblages is the numerical dominance of a few species, most members of the community being low in numbers.

**Figure 7.**
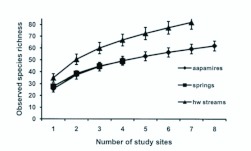
Sample-based species accumulation curves of semiaquatic flies in aapa mires, springs and headwater streams, means and SDs (Mao-Tau method) are shown for the each number of study sites. High quality figures are available online.

**Figure 8.**
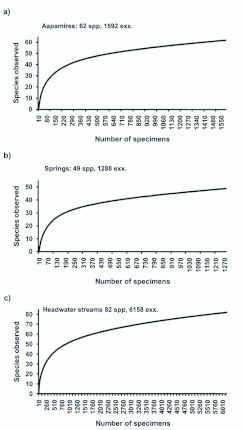
Individual based rarefaction curves of semiaquatic flies in a) aapa mires, b) springs and c) headwater streams. Material is combined for the respective habitat types. High quality figures are available online.

Accumulation of species richness (or cumulative number of species) was highest in the headwater streams (Figure 7). The cumulative number of species in springs would probably have risen, had there been greater sampling effort (only four sites studied). According to rarefaction, in all habitat types the number of observed species rises rapidly in the beginning, but levels-off after ca. 50 % of the species are captured (Figure 8a–c). For example, a standardized sample size of 800 specimens would have captured 53 (85 % of the observed number of species), 44 (90 %) and 48 (59 %) species for aapa mires, springs and headwater streams, respectively.

**Table 6.**
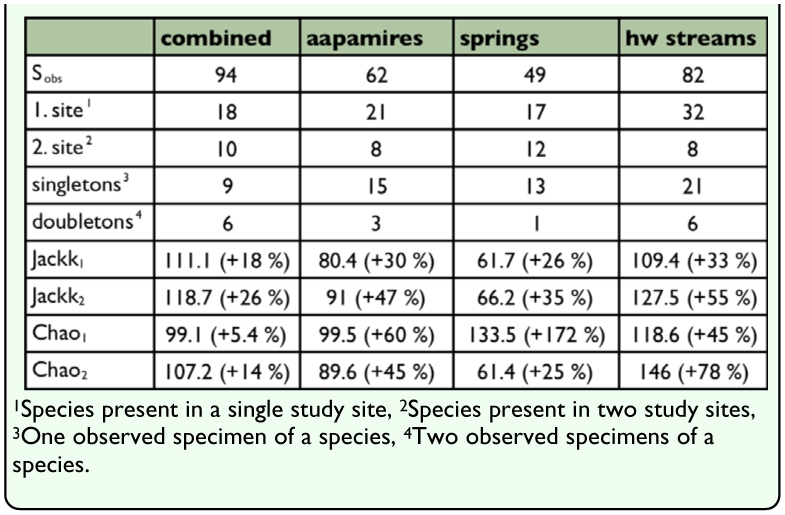
Observed (Sobs) and estimated species richness of semiaquatic flies in the combined material (n= 19) and amongst the habitat types. Estimated values are based on non-parametric estimators (Jackknife1, Jackknife2, Chao1, Chao2), percentual values are the additions of estimators to the observed values.

According to Jackknife and Chao estimators, the observed species richnesses among the habitat types were 25–172 % lower than estimated number of species (Table 6). For the combined material of the study area (19 study sites, 94 spp), the observed number of species was 5.4–26 % lower than estimated number of species.

### Frequency and abundance of the most common semiaquatic flies, rare species and preliminary assessment of the conservation value of the habitat types

**Figure 9.**
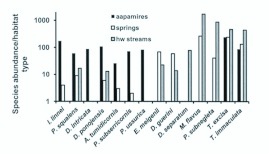
Abundance (number of specimens) of 14 semiaquatic fly species in the studied habitat types. For example, *ldioptera linnei* and *Phylidorea squalens* were most abundant in aapa mires but were also present in other habitat types. *Molophilus flavus* and *Parabazarella subnegleta* were very numerous in headwater streams and springs but were absent from aapa mires. *Tipula excisa* and *Tricyphona Immaculata*, eurytopic craneflies, were rather abundant in all habitat types. High quality figures are available online.

The most common (frequency >75 % across all study sites) species of the studied material were *Tipula excisa* Schummel (100 %), *Tipula subnodicornis* Zetterstedt (100 %), *Tricyphona immaculata* (Meigen) (95 %), *Phylidorea squalens* (Zetterstedt) (84 %), *Dicranomyia distendens* Lundström (89 %), *Ptychoptera minuta* Tonnnoir (84 %), *Tipula limbata* Zetterstedt (84 %), *Pedicia rivosa* (L.) (79 %) and *Idioptera pulchella* (Meigen) (78 %); only one out of nine species was not a cranefly. As one can expect on the basis of community analyses, the abundances and frequencies of the species were highly variable between different habitat types. For example, *Idioptera linnei* Oosterbroek, *Prionocera subserricornis* Zetterstedt and *Pneumia ussurica* Wagner were typical inhabitants of aapa mires (Figure 9). No such abundant species (total number of specimens >30) displayed fidelity to, or were found exclusively from springs. However, *Euphylidorea meigenii* (Verrall) and *Dicranota guerini* Zetterstedt were more numerous in springs than either in aapa mires or headwater streams (Figure 9). Headwater streams harbored several species that were not present elsewhere (e.g. *Dicranophragma separatum* [Walker]) or the species occurred abundantly in springs and headwater streams (e.g. *Molophilus flavus* Goetghebuer, *Parabazarella subnegleta* [Tonnoir], Figure 9). *Tipula excisa* and *Tricyphona immaculata*, among the most eurytopic species, were abundant in the all habitat types (Figure 9).

A total 13 semiaquatic fly species were classified as noteworthy, i.e. the species are considered to indicate conservation value of their habitats (Appendix 1). These species are either red-listed, that is, threatened or near threatened in the Finnish Red-Data book or listed as national responsibility species of Finland (see e.g. Salmela 2009, Penttinen et al. 2010). [Additional information dealing with these species is available from the author]. Most of the occurrences of these species were encountered in aapa mires (36 records), followed by headwater streams (24) and springs (19); no statistical difference between the habitat types was found (χ^2^=5.88, df=2, *p*=0.053). There was no difference in the mean number of noteworthy species in the habitat types (aapa mires 4.5, springs 3.4 and headwater streams 4.8, ANOVA F=0.6, df=2, *p*=0.56).

## Discussion

### Community structure, classification of habitat types and assemblage concordance of semiaquatic flies and plants

The semiaquatic fly assemblages of aapa mires were clearly differentiated from those of springs and headwater streams, but no difference was found between the two latter habitat types. This result is rather surprising, since springs deviate from other wetland habitats due to their constant temperature and discharge of water. There are several crenobiontic (spring dependent) and crenophilous (favoring springs) semiaquatic fly species, especially in southern boreal Finland (e.g. Salmela *et al*. 2007) and Central Europe (e.g. Reusch and Hohmann 2009). In the subalpine fell areas of Fennoscandia, headwater streams may often be spring-fed, but the ones studied here displayed higher summer water temperatures than the springs (see Table 2) and received their water from up-stream lakes or mires. Thus, it can be safely concluded that the studied headwater streams were not appreciably influenced by the groundwater. In a study conducted in the Finnish northern boreal zone, spring semiaquatic fly communities did not form their own, plain cluster group or assemblage type, but showed compositional similarity to rich fens and headwater streams (Salmela 2008). It is very likely that the semiaquatic fly fauna of northern boreal region is characterized by low number of crenobiontic species. Such almost strictly spring-dwelling species are *Rhabdomastix parva* (Siebke) *Tipula fendleri* Mannheims and *Pneumia pilularia* (Tonnoir) (Salmela 2008, J. Salmela, unpubl.). Crenophilous species (e.g. *Dicranomyia caledonica* Edwards, *D. stylifera* Lackschewitz and *Dicranota guerini*) are usually abundant or occur frequently in springs, but are also common in lotic waters or rich fens. Hence, in this light, it is not so puzzling that semiaquatic fly assemblages of springs in the subalpine ecoregion are not so distinctive.

In this study, only two taxa were exclusively present in springs (*Dicranota bimaculata* [L.] and *Dicranomyia* (*Melanolimonia*) sp, female, most likely *D. caledonica* or *D. stylifera*). Abundant and/or frequent members of the spring assemblages were also abundant in headwater streams (e.g. *Molophilus flavus, Dicranota exclusa, Parabazarella* *subnegleta*). Quite evidently, several springdwelling semiaquatic flies are lotic species that are able to complete their life cycles in thermally constant springs. Based on the author's personal assessment, a part of the northern boreal spring-dwelling semiaquatic flies are calciphilous, in other words, prefer sites with high pH value and specific conductivity. The springs of the study area were not calcareous (assessed from the plant species composition, pH and bedrock composition), and thus, the lack of suitable habitat conditions may explain the absence of such species in this study. Calciphilous northern species are at least *D. caledonica, D. stylifera, Pneumia pilularia* and *Rhabdomastix parva*; the last mentioned species is perhaps restricted to the Caledonian mountains in Finland (Salmela 2008). Based on the results of this study and previous studies, the semiaquatic fly community composition of springs in the northern boreal zone is mostly “nested” (see e.g. Cutler 1998, Ulrich and Gotelli 2007) with surface fed streams and the species richness of springs is usually lower compared to that of headwater streams. On the other hand, influence of upwelling groundwater is usually in connection with high species richness of semiaquatic flies in small lotic waters and mires (Salmela 2008).

In the present study the comparison of semiaquatic fly faunas between ecologically rather different wetland habitats was seen meaningful, because the variation in species composition and richness in such a small spatial scale (ca. 225 km^2^) has not been investigated before in Fennoscandia. It is quite clear that the habitat type can be used as a rough predictor of the semiaquatic fly assemblage, although there is more or less between-habitat overlap in species composition. The spatial structure, i.e. geographical distance between the study sites was not strongly associated with their faunistic resemblance. Instead, much stronger association was noted with the environmental distance and species composition. Despite the several distinguishing characteristics, and the differences between the aapa mires, springs and headwater streams, the studied habitats and their semiaquatic fly assemblages do form a wetland gradient, which is mainly influenced by conditions and resources of peatlands, lotic waters and their riparian zones; altitude is part of this multidimensional habitat space. In addition to environmental factors, biotic interactions (competition, predation, parasitism) are also central determinants of community composition (Morin 1999). It is unfortunate that knowledge about these biotic factors and their importance for community level organization among semiaquatic flies is almost negligible. According to an elegant study by Freeman (1967), physical properties of environment (e.g. substrate, moisture) are likely to be the most important drivers of the soil-dwelling *Tipula* communities, although (intraspecific) larval competition for space may occur and cause high mortality. Freeman (1967) stresses that the coexistence of several *Tipula* species within small spatial scale (some hundreds of square meters) is explainable by the fine-scale niche separation between the species, i.e. differential use of microhabitats. Further, temporal separation (phenology) between closely related species perhaps lessens the probability of interspecific competition (Freeman 1967).

According to Mantel tests, plant species composition explained very well the variation in resemblance of semiaquatic fly assemblages. Mantel test values and associated coefficients of determination showed high matrix concordance between semiaquatic flies and higher plants and between semiaquatic flies and combined plant material; the concordance between bryophytes was much weaker. Indeed, higher plant and combined plant species composition (R^2^: 37.5–40.4 %) explained much better the variation in semiaquatic fly species composition than either geographical distance (R^2^=3.3 %) or environmental variables (R^2^= 15.6 %). Thus, the results of this study are in accordance with Schaffers *et al*. (2008), who claimed that arthropod assemblages are best predicted by plant species composition (their study encompassed spiders and several terrestrial insect groups in meadow habitats). It has traditionally been considered that insect communities are determined by environmental conditions and (physical) vegetation structure. However, plant species composition is largely shaped by environmental conditions and summarizes these conditions that may fluctuate over time, and plants also modify their own environment (Schaffers *et al*. 2008). The integrating nature (referring to conditions, physical structure and microhabitats) of plant communities is apparently important for arthropods in all trophic levels both directly (obligate herbivores) and indirectly (e.g. soil dwellers and epigean predators). To conclude, in the subalpine ecoregion the nematoceran and plant assemblages are co-varying according to the same wetland gradients. As pointed out by Schaffers *et al*. (2008), this co-variation is partly indirect, probably due to a similar pattern of response to underlying environmental variables, and partly direct, due to the obligate associations between biotic groups. For example, in the present study the relatively common *Phalacrocera replicata* (Cylindrotomidae) eats and dwells among aquatic bryophytes (Peus 1952), larvae of the peatland species *Idioptera linnei* (Limoniidae) inhabit *Sphagnum* mosses (Boardman 2004) and adult flies may feed on liquids secreted by woody plants (Stubbs 2005) or flower nectars (Chernov and Lantsov 1992).

Community concordance between different taxonomic groups may be rather weak (Similä et al. 2006; Carlisle et al. 2008; Davis et al. 2008; Mykrä et al. 2008) and significant only after the environmental gradients (e.g. location of study sites in multiple ecoregions) are rather long (Paavola et al. 2006). The relatively low concordance may reflect divergent responses of different taxonomic groups to the prevailing environmental factors (Virtanen et al. 2009). On the other hand, the positive cross-taxon congruence may be relatively high, although the same groups would not display correlation in species richness (Su et al. 2004). One should bear in mind that, despite the close geographical proximity of the study sites, the environmental gradients in this study were long, because a selection of such varied habitat types was included. Usually community concordance has been studied within a single habitat, such as streams (Paavola et al. 2006), ponds (Bilton et al. 2006) and springs (Virtanen et al. 2009). As noted above, plant species composition, especially that of higher plants, seems to be the best predictor of semiaquatic fly assemblage structure. This result has importance in nature conservation and monitoring, whether assemblages of a single taxonomic group are to be used as a surrogate for other groups. However, more studies are needed (i) in a larger spatial scale and (ii) within a habitat type before sound recommendations about the potential congruence between higher plants and semiaquatic flies can be made.

Although semiaquatic fly assemblages of small lotic waters (springs and headwater streams) were clearly distinguishable from those of aapa mires, there was a notable overlap in species composition between the habitat types. For example, several peatland-dwelling species were present in headwater streams (e.g. *Phylidorea squalens, Dicranomyia terraenovae* Alexander, *Prionocera ringdahli* Tjeder); these species have most probably spent their immature stages in mire vegetation in the vicinity of the traps. Further, eurytopic generalist species (e.g. *Tipula excisa, Tricyphona immaculata*), which are common in a multitude of moist habitats, were encountered in all habitat types. Hence, the observed species overlap between the habitat types is most likely explainable by two factors: (i) small lotic waters are not extremely clear-cut but part of the wetland gradient and (ii) high frequency of occurrence of generalist species increase the between site similarity. The trapping method used in this study (Malaise) is non-selective; the trap collects insects from a wider area than emergence traps, for example. Nevertheless, the Malaise trap is passive, it does not attract insects in similar manner as the light trap. Based on the author's experience from >400 Malaise trapping sites, it can be estimated that the vast majority of the collected specimens have spent their immature life cycle within or in the near vicinity of the 10 × 30 m study plots. Larger adult flies, especially craneflies, probably have good potential for dispersal, but are usually found in close vicinity to their larval habitat (Freeman 1968).

### Rank-abundance distributions and patterns in species richness

The rank-abundance distribution patterns of semiaquatic fly assemblages in the different habitat types could perhaps be classified as truncated log-normal type (Magurran 2004; Ulrich and Ollik 2005), although no tests of goodness-of-fit to any statistical distribution model was performed. This kind of rankabundance distribution is commonly recorded across different biomes and taxonomic groups (Ulrich and Ollik 2005). Log-normal distribution is characterized by the dominance of one or a few species, most of the species being relatively scarce, i.e. in low numbers. In spite of the seemingly similar rank-abundance patterns in this study, the habitat types were differentiated by the proportion of the most numerous species. The mean proportion of the most abundant species was lowest in aapa mires, higher in springs and headwater streams, in ascending order. In the same vein, the Simpson diversity index values were highest in aapa mires, lower in the springs and headwater streams. In other words, semiaquatic fly assemblages of aapa mires were relatively even, and species abundances were more skewed amongst springs and headwater streams. Nevertheless, rankabundance distribution in a pristine aapamire can be heavily skewed. A sample of >500 specimens belonging to seven species were collected from a carpet-lawn level, poor fen in the northern boreal zone, Kittilä (Finland): tipulid species *Tipula subnodicornis* accounted for 91 % of the total number of specimens (Salmela 2008). In contrast, intermediate rich and rich fens (as the aapa mires of this study) are more species rich than poor fens (Salmela 2004, 2008) and possibly also display higher evenness.

Cumulative species richness was clearly highest in headwater streams, in other words its species pool was richer than in aapa mires or springs. As there were only four springs studied, the cumulative species richness would probably had been higher had more sites been sampled. Actually, none of the species accumulation curves did not reach an asymptote, and it is likely that species belonging to the local species pool were unrecorded (see below). Individual based species accumulation curves (rarefaction) indicate that observed species richness rose rapidly at first but leveled-off after ca. 50 % of the species were captured. It is hard to give any recommendations about the representative sampling effort (number of traps/study sites or collected specimens). One should consider what is adequate to fulfill the aims of the study: is it sufficient to collect the most common or abundant species, which would allow beta diversity comparison, or is the aim to collect also the rarest members of the assemblage? In species rich taxonomic groups, the probability of catching a rare species is higher the larger the collected sample is (Martikainen and Kouki 2003). Furthermore, the accumulation of rare species is lower than that of common species. A study performed in only one field season does not possibly record all low abundance species (Martikainen and Kaila 2004).

According to non-parametric species richness estimators, there were unseen species in all studied habitat types, perhaps more so in headwater streams than aapa mires or springs. These estimates are, of course, just indications, not absolute facts, because the number of study sites for each habitat type was rather low. Perhaps the best estimate is obtained by combining the material gathered from 19 sites (94 observed species), which indicates that 5.4–26 % of the species in the study area were not collected. A total of 192 semiaquatic fly species are recorded from the biogeographical province of *Lapponia enontekiensis* (J. Salmela, unpublished data), and thus there is a high probability of encountering more species from the study area than were sampled in the present study.

### General notes on the observed semiaquatic fly fauna and conservation value assessment

The most frequently encountered semiaquatic fly species of the studied material are widespread and common inhabitants of various wetlands in Finland. The only exception is *Tipula excisa*, a northern species, which is not present south of the middle boreal zone (J. Salmela, unpublished observation). Almost 20 % of the semiaquatic fly species were rather rare, since they were collected from only one site. As already observed from the rank-abundance distributions, there were rather few very abundant species: seven of the most abundant species (>350 specimens) accounted for 65 % of the total number of collected specimens. Most of the abundant or relatively numerous species showed rather high fidelity for a certain habitat type (see Figure 10); in the light of community analyses, this is obvious.

The most remarkable faunistic record of this study was a limoniid, *Dicranomyia intricata* Alexander, a species which have hitherto been found from the northern Baltic in Finland, some 600 km south from the study area (J. Salmela, unpublished observation). There is one old record from North Sweden, Abisko, a collection of a holotype male of *D. suecica* (Nielsen 1953), which is a synonym of *D. intricata*. In the Palaearctic region, the species is not known to occur outside Finland and Sweden (Oosterbroek 2010). The species was described from the Nearctic, Canada; apparently the species occurs there in boreal mires (Alexander 1927). *Dicranomyia intricata* was quite numerous in two close lying aapa mires (Tomuttirova I and II), in three other sites only singletons were present (aapamire, spring and headwater stream). Other rare or otherwise notable species were e.g. *Dicranomyia Mensis* (Tjeder) (endemic to Fennoscandia), *Prionocera abscondita* Lackschewitz (internationally rare, arctic species), *Prionocera woodorum* Brodo (rare and poorly known, northern species) and *Tipula laccata* Lundström and Frey (rare, northern).

Thirteen species, 14 % of the total number of observed species, were classified as noteworthy. These species will be threatened or near threatened in the Red-Data book of Finland, or the species are National Responsibility Species of Finland (Penttinen et al. 2010, J. Salmela, unpublished data). Although no statistically significant differences of the occurrences of such species between habitat types were noted, aapa mires (36) harbored more occurrences than headwater streams (24) or springs (19). Most of these noteworthy species are principally mire species (8 spp), four species could be classified inhabitants of headwater streams or alpine wetlands and only species is crenophilous. Thus, it is not surprising that aapa mires display high conservation value as habitats for semiaquatic flies. From an international view point, aapa mires are important because many mire species having viable populations with no current threat in Finland (e.g. *Priocera chosenicola* Alexander, *P. pubescens* Loew, *Idioptera linnei*) are extremely rare or threatened in the other parts of Europe (Boyce 2004; Martinovský and Barták 2005).

## Figures and Tables

**Appendix 1:**
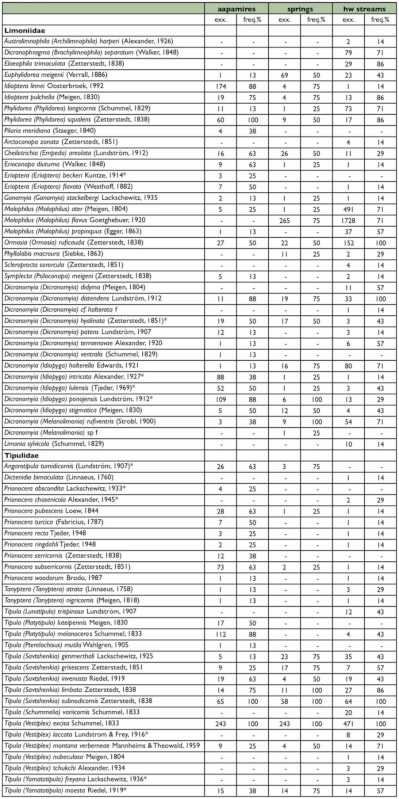
Semiaquatic flies and their abundances (total number of specimens) and frequencies (%) among the studied habitat types. * indicates noteworthy species (red-listed in 2010 or national responsibility species). Aapamires n=8, springs n=4, headwater streams n=7.

**Appendix 2.**
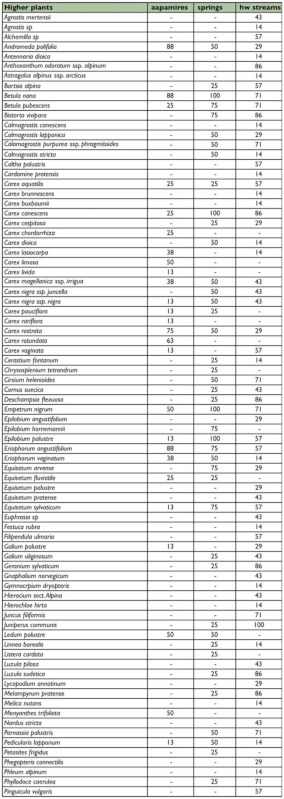
Higher plants and bryophytes and their frequencies (%) amongst the studied habitat types. Species are in alphabetical order. Aapamires n=8, springs n=4, headwater streams n=7.

## Abbreviations

**ANOSIM**,: analysis of similarity test
**MRPP**,: multi-response permutation procedure
**NMS**,: nonmetric multidimensional scaling
